# Perioperative Risks of Concurrent Pancreatic and Hepatic Resections for Neuroendocrine Tumors

**DOI:** 10.1002/jso.70352

**Published:** 2026-08-06

**Authors:** Amir Ebadinejad, Ashrita Raghuram, Sophia Xiao, Ethan Angle, Hassan Aziz

**Affiliations:** ^1^ Department of Surgery University of Iowa Hospitals and Clinics Iowa City Iowa USA; ^2^ Department of Surgery University of Maryland Baltimore Maryland USA; ^3^ Carver College of Medicine University of Iowa Iowa City Iowa USA; ^4^ Department of Surgery Mayo Clinic Rochester Minnesota USA

**Keywords:** ACS‐NSQIP, hepatectomy, neuroendocrine tumor, pancreatectomy, short‐term outcomes

## Abstract

**Background and Objectives:**

Pancreatic neuroendocrine tumors (pNETs) account for 1%–2% of pancreatic tumors, with an increasing incidence. The safety of concurrent pancreas‐liver resections remains uncertain. This study evaluated the national outcomes of concurrent pancreatectomy and liver‐directed surgery for pNETs.

**Methods:**

ACS‐NSQIP (2019–2023) was queried for adult patients who underwent pancreatectomy for pNETs. Concurrent liver‐directed procedures were identified by CPT code and classified as hepatectomy or ablation alone; patients were grouped as isolated pancreatectomy, concurrent hepatectomy, or concurrent ablation alone. Pre‐, intra‐, and postoperative characteristics were compared using inferential statistics. Multivariable logistic regression identified predictors of 30‐day morbidity, readmission, reoperation, and mortality, with Firth penalized likelihood used for rare outcomes.

**Results:**

Of 4794 patients, 4587 (95.7%) underwent isolated pancreatectomy, 172 (3.6%) underwent concurrent hepatectomy, and 35 (0.7%) underwent concurrent ablation alone. Concurrent hepatectomy was parenchymal‐sparing in 163 of 172 patients (94.8%); only nine patients underwent a major hepatectomy. Overall morbidity was similar across groups (38.8%, 45.9%, and 42.9%; *p* = 0.154), as was serious morbidity (28.7%, 27.9%, 22.9%; *p* = 0.730) and 30‐day mortality (1.0%, 0.6%, 0%). On multivariable analysis, neither concurrent hepatectomy (OR 1.02, 95% CI 0.71–1.48) nor concurrent ablation (OR 0.94, 95% CI 0.46–1.93) was associated with morbidity. Pancreatoduodenectomy was associated with morbidity (OR 2.38, 95% CI 2.05–2.76), reoperation (OR 2.16, 95% CI 1.52–3.08), and mortality (OR 2.50, 95% CI 1.39–4.50), and a minimally invasive approach was associated with lower morbidity (OR 0.69, 95% CI 0.60–0.80).

**Conclusions:**

Concurrent liver‐directed surgery was not associated with an increase in 30‐day morbidity or mortality after pancreatectomy for pNETs. These findings support the safety of concurrent hepatic resection in appropriately selected pNET patients.

## 1 Introduction

Pancreatic neuroendocrine tumors (pNETs) originate from pancreatic islet cells and are a rare type of heterogeneous pancreatic neoplasm [1]. pNETs are categorized into functional and non‐functional tumors depending on their ability to secrete hormones, such as gastrin, glucagon, vasoactive intestinal peptide, and somatostatin [2]. While rare, with an incidence of less than one case per 100 000 per year, the number of pNET cases and overall survival have been increasing over the past few decades [1, 2]. Five‐year survival for well‐differentiated, low‐grade pNETs exceeds 60%, whereas median survival falls substantially once distant metastases are present [3]. Often, pNET patients present at the time of diagnosis with high rates of metastatic disease, with estimates of up to 80% [4, 5, 6]. Due to their heterogeneous nature and few effective systemic therapies, treatment of pNETs remains a challenge [4].

Surgery plays a key role in the management of pNETs and can range from complete resection to cytoreductive surgery in patients with pNETs and resectable metastases [5, 6]. The liver is the most common site of metastasis; thus, liver‐directed surgery is often performed in addition to primary resection [7]. Complete macroscopic clearance of hepatic metastatic disease is achievable in only a minority of patients [6, 7, 8]. Tumor recurrence is common. However, 5‐year median survival rates approaching 85% indicate that aggressive surgical resection is beneficial, especially in localized and locally advanced pNETs [7, 9]. Tumor resection is often performed in patients with functional pNETs for symptom control.

Resection of the primary lesion and hepatic metastases can vary depending on the type of liver‐directed procedure (minor or major hepatectomy vs. ablation or cytoreductive surgery) and timing (concurrent resection or in a staged manner). While no randomized studies have compared surgical resection to non‐surgical management, survival outcomes for patients undergoing partial hepatectomy have been shown to surpass those treated with chemoembolization [8]. Cytoreductive surgery improves symptom management, survival, and quality of life [7]. Kone et al. recently reported that patients undergoing simultaneous pancreatic and hepatic resection experienced worse postoperative outcomes than patients undergoing isolated hepatectomy, while having a morbidity profile broadly similar to that of pancreatectomy alone [10]. However, there is a paucity of literature that differentiates the safety of various concurrent pancreatic and liver‐directed techniques in the perioperative period.

This study evaluated the perioperative outcomes of patients undergoing concurrent pancreatic and liver‐directed surgery for pNETs. Understanding the impact of hepatic procedures on recovery and comparing different resection strategies may guide surgical planning and improve patient outcomes.

## 2 Materials and Methods

### 2.1 Data Source

We performed a retrospective cohort study using the American College of Surgeons National Surgical Quality Improvement Program (ACS‐NSQIP) Procedure‐Targeted Pancreatectomy Participant Use Files from 2019 to 2023. The NSQIP prospectively collects standardized clinical data on preoperative risk factors, intraoperative variables, and 30‐day postoperative outcomes from participating hospitals in the United States.

### 2.2 Study Population

Adult patients (≥ 18 years) who underwent pancreatectomy were identified from the Procedure‐Targeted Pancreatectomy files. The cohort was defined by histology recorded on the pancreatectomy‐targeted variables rather than by ICD diagnosis codes: patients were eligible if malignant histology was recorded as neuroendocrine‐functioning or neuroendocrine‐nonfunctioning, or if benign histology was recorded as neuroendocrine without metastases. This yielded 4860 patients. We excluded 66 patients whose only liver‐directed procedure was captured by an unlisted CPT code (47379, 47399), because the extent of resection cannot be determined from an unlisted code. The primary analytic cohort comprised 4794 patients.

### 2.3 Definition of Concurrent Liver‐Directed Procedures

Concurrent procedures were identified from the other‐procedure and concurrent‐procedure CPT fields by CPT code. Groups were assigned hierarchically as resection, then ablation, then biopsy. Major hepatectomy was defined by CPT 47122, 47125, or 47130; minor (parenchymal‐sparing) hepatectomy by CPT 47120; and ablation by CPT 47370, 47371, 47380, 47381, 47382, or 47383. Wedge liver biopsy (CPT 47100) were not treated as liver‐directed therapeutic procedures. The complete code list is provided in Supporting Information S2: Table 1.

Only nine patients underwent a formal major hepatectomy. Major and minor hepatectomy were therefore combined into a single concurrent hepatectomy group for regression modeling, and major hepatectomy is reported descriptively. The three analytic groups were isolated pancreatectomy (*n* = 4587), concurrent hepatectomy (*n* = 172), and concurrent ablation alone (*n* = 35).

### 2.4 Variables and Outcomes

Preoperative characteristics included age, sex, body mass index, comorbidities, functional status, preoperative albumin and total bilirubin, and receipt of chemotherapy within 90 days of surgery. Intraoperative variables included procedure type (distal pancreatectomy, pancreatoduodenectomy, or other), operative approach, and operative time.

The primary outcome was a composite of any 30‐day morbidity, constructed only from NSQIP fields collected in every study year. Renal insufficiency is not collected in the 2021 file and was therefore excluded from the composite, while acute renal failure and postoperative dialysis, which are collected in all years, were retained. A secondary composite of serious morbidity excluded superficial surgical site infection, urinary tract infection, transfusion, and delayed gastric emptying. Pancreatic fistula and postoperative acute pancreatitis were defined as ISGPS grade B or C; biochemical leak and grade A pancreatitis were not counted as events. Post‐pancreatectomy hemorrhage (collected 2021–2023) and postoperative acute pancreatitis (collected 2019–2020) are reported against their year‐restricted denominators. Additional outcomes were 30‐day mortality, readmission, and reoperation. Outcome definitions and their availability by file year are given in Supporting Information S2: Table 3.

### 2.5 Statistical Analysis

Continuous variables are reported as median with interquartile range and compared with the Kruskal–Wallis test. Categorical variables are summarized as counts and percentages and compared using the chi‐square test, the Fisher exact test for 2 × 2 tables with sparse cells, or a Monte Carlo Fisher–Freeman–Halton test with 20 000 resamples for larger sparse tables. Every proportion is reported with its own denominator, and the extent of missing data for each variable is reported in Supporting Information S2: Table 4.

Covariates for the multivariable models were prespecified on clinical grounds rather than selected by a data‐driven procedure, and comprised age, sex, body mass index, ASA class, functional status, preoperative albumin and total bilirubin, chemotherapy within 90 days, type of pancreatic resection, operative approach, pathologic M stage as a proxy for metastatic burden, neuroendocrine functional status, and file year. Because operative approach, laboratory values, and M stage are incompletely recorded in NSQIP, the primary models retained the full cohort by entering missingness as an explicit indicator or category; complete‐case and multiply imputed models (m = 10, pooled by Rubin's rules) are reported as sensitivity analyses. Reoperation and mortality were modeled with Firth penalized likelihood and profile‐likelihood confidence intervals given the small number of events in the ablation group, and the mortality model was limited to five parameters. The study‐group term was tested as a block by likelihood‐ratio test on two degrees of freedom. Prespecified sensitivity analyses comprised inclusion of the unlisted liver CPT codes within the hepatectomy group, restriction to patients with documented M1 disease, and the complete‐case and multiply imputed models. All analyses were performed using Stata/SE 19.5 (StataCorp LLC, College Station, TX) and Python 3.12 (SciPy 1.17, scikit‐learn 1.8). Statistical significance was defined as *p* < 0.05.

### 2.6 Ethical Considerations

NSQIP data were de‐identified and collected for quality improvement purposes; therefore, this study was exempt from institutional review board approval in accordance with federal regulations.

## 3 Results

### 3.1 Patient and Procedural Characteristics

Among 4794 patients undergoing pancreatectomy for a pNET, 207 (4.3%) underwent a concurrent liver‐directed procedure: 172 a concurrent hepatectomy and 35 an ablation alone. Concurrent hepatectomy was almost entirely parenchymal‐sparing; 163 of 172 patients (94.8%) underwent a partial or wedge resection, and only nine patients in the entire 5‐year national cohort underwent a hemihepatectomy or trisegmentectomy.

Documented M1 disease was present in 119 of 172 patients undergoing concurrent hepatectomy (69.2%) and 24 of 35 undergoing ablation (68.6%), compared with 160 of 4587 (3.5%) undergoing isolated pancreatectomy. Baseline comorbidity was broadly similar across groups (Table 1). Median operative time was 247 min for isolated pancreatectomy and 346 min for both concurrent hepatectomy and concurrent ablation (Table 2). A minimally invasive approach was used less often when a liver‐directed procedure was performed.

**Table 1:** Preoperative characteristics of patients undergoing pancreatectomy for pancreatic neuroendocrine tumors, stratified by concurrent liver‐directed procedure.

Variable	Isolated pancreatectomy (*n* = 4587)	Concurrent hepatectomy (*n* = 172)	Concurrent ablation (*n* = 35)	*p* value	Missing, n (%)
Age, years — median [IQR]	61.0 [51.0–69.0] (*n* = 4587)	56.5 [47.8–65.0] (*n* = 172)	52.0 [44.5–63.0] (*n* = 35)	< 0.001	0 (0.0%)
Male sex	2482/4587 (54.1%)	92/172 (53.5%)	24/35 (68.6%)	0.227	0 (0.0%)
Body mass index, kg/m^2^ — median [IQR]	29.4 [25.4–33.8] (*n* = 4558)	28.2 [24.5–33.6] (*n* = 171)	27.3 [25.1–31.5] (*n* = 35)	0.035	30 (0.6%)
ASA class III–V	3414/4583 (74.5%)	134/172 (77.9%)	27/35 (77.1%)	0.566	4 (0.1%)
Dependent functional status	28/4583 (0.6%)	1/172 (0.6%)	0/35 (0.0%)	1.000	4 (0.1%)
Current smoker	459/4587 (10.0%)	23/172 (13.4%)	4/35 (11.4%)	0.345	0 (0.0%)
Diabetes mellitus	1044/4587 (22.8%)	37/172 (21.5%)	5/35 (14.3%)	0.459	0 (0.0%)
Hypertension requiring medication	2316/4587 (50.5%)	71/172 (41.3%)	14/35 (40.0%)	0.029	0 (0.0%)
Severe COPD	82/4587 (1.8%)	4/172 (2.3%)	1/35 (2.9%)	0.921	0 (0.0%)
Congestive heart failure	68/4587 (1.5%)	0/172 (0.0%)	1/35 (2.9%)	0.257	0 (0.0%)
Preoperative albumin, g/dL — median [IQR]	4.2 [3.9–4.5] (*n* = 3775)	4.2 [4.0–4.5] (*n* = 150)	4.0 [3.9–4.3] (*n* = 34)	0.178	835 (17.4%)
Preoperative bilirubin, mg/dL — median [IQR]	0.5 [0.3–0.7] (*n* = 3883)	0.5 [0.3–0.7] (*n* = 153)	0.5 [0.4–0.9] (*n* = 34)	0.362	724 (15.1%)
Chemotherapy within 90 days	163/4560 (3.6%)	25/169 (14.8%)	6/35 (17.1%)	< 0.001	30 (0.6%)
Pathologic M stage				< 0.001	0 (0.0%)
M0/Mx	2912/4587 (63.5%)	27/172 (15.7%)	7/35 (20.0%)		
M1	160/4587 (3.5%)	119/172 (69.2%)	24/35 (68.6%)		
Unknown/not recorded	1515/4587 (33.0%)	26/172 (15.1%)	4/35 (11.4%)		
Neuroendocrine histology				0.001	0 (0.0%)
Functioning	901/4587 (19.6%)	40/172 (23.3%)	11/35 (31.4%)		
Non‐functioning	3183/4587 (69.4%)	127/172 (73.8%)	24/35 (68.6%)		
Benign/no malignant histology	503/4587 (11.0%)	5/172 (2.9%)	0/35 (0.0%)		
NSQIP PUF year				0.318	0 (0.0%)
2019	877/4587 (19.1%)	35/172 (20.3%)	7/35 (20.0%)		
2020	828/4587 (18.1%)	42/172 (24.4%)	5/35 (14.3%)		
2021	930/4587 (20.3%)	29/172 (16.9%)	7/35 (20.0%)		
2022	975/4587 (21.3%)	41/172 (23.8%)	8/35 (22.9%)		
2023	977/4587 (21.3%)	25/172 (14.5%)	8/35 (22.9%)		

*Note:* Data are n/N (%), where N is the number of patients with the variable recorded, or median [interquartile range]. Continuous variables were compared with the Kruskal–Wallis test; categorical variables with the chi‐square test, Fisher exact test, or a Monte Carlo Fisher–Freeman–Halton test where cell counts were sparse.

Abbreviations: COPD, chronic obstructive pulmonary disease; NSQIP, National Surgical Quality Improvement Program.

**Table 2:** Operative characteristics, stratified by concurrent liver‐directed procedure.

Variable	Isolated pancreatectomy (*n* = 4587)	Concurrent hepatectomy (*n* = 172)	Concurrent ablation (*n* = 35)	*p* value	Missing, n (%)
Pancreatic resection				0.037	0 (0.0%)
DP	2492/4587 (54.3%)	90/172 (52.3%)	20/35 (57.1%)		
Whipple	1369/4587 (29.8%)	67/172 (39.0%)	10/35 (28.6%)		
Other	726/4587 (15.8%)	15/172 (8.7%)	5/35 (14.3%)		
Operative approach				< 0.001	496 (10.3%)
Open	2122/4111 (51.6%)	132/156 (84.6%)	26/31 (83.9%)		
MIS	1989/4111 (48.4%)	24/156 (15.4%)	5/31 (16.1%)		
Operative time, min — median [IQR]	247.0 [172.0–348.0] (*n* = 4587)	346.0 [247.8–446.0] (*n* = 172)	346.0 [233.5–445.0] (*n* = 35)	< 0.001	0 (0.0%)
Length of stay, days — median [IQR]	5.0 [4.0–8.0] (*n* = 4514)	7.0 [5.8–9.0] (*n* = 168)	8.0 [5.5–11.5] (*n* = 35)	< 0.001	77 (1.6%)

*Note:* Data are n/N (%), where N is the number of patients with the variable recorded, or median [interquartile range]. Continuous variables were compared with the Kruskal–Wallis test; categorical variables with the chi‐square test, Fisher exact test, or a Monte Carlo Fisher–Freeman–Halton test where cell counts were sparse.

### 3.2 Postoperative Outcomes

Any 30‐day morbidity occurred in 1780 of 4587 patients (38.8%) after isolated pancreatectomy, 79 of 172 (45.9%) after concurrent hepatectomy, and 15 of 35 (42.9%) after concurrent ablation (*p* = 0.154). Serious morbidity occurred in 28.7%, 27.9%, and 22.9%, respectively (*p* = 0.730). Thirty‐day mortality was 1.0%, 0.6%, and 0%. Readmission (18.7%, 16.3%, 14.3%) and reoperation (3.8%, 3.5%, 5.7%) did not differ across groups. Clinically significant pancreatic fistula occurred in 18.5%, 15.9%, and 14.3% (*p* = 0.555). Observed rates of the primary 30‐day outcomes for each group, with 95% confidence intervals, are shown in Supplementary Figure S2.

Bleeding requiring transfusion was the single complication that differed between groups, occurring in 8.6% of isolated pancreatectomy patients, 19.8% after concurrent hepatectomy, and 22.9% after concurrent ablation (*p* < 0.001). Post‐pancreatectomy hemorrhage, assessed in the 2021–2023 files, occurred in 6.2%, 7.4%, and 26.1% (95% CI 10.2%–48.4%), respectively (*p* < 0.001), although the ablation group contributed only 23 patients to that comparison, and this estimate should be interpreted with caution given the wide confidence interval (Table 3; Supporting Information S1: Figure 3).

**Table 3:** Thirty‐day postoperative outcomes, stratified by concurrent liver‐directed procedure.

Variable	Isolated pancreatectomy (*n* = 4587)	Concurrent hepatectomy (*n* = 172)	Concurrent ablation (*n* = 35)	*p* value	Missing, n (%)
Any morbidity (composite)	1780/4587 (38.8%)	79/172 (45.9%)	15/35 (42.9%)	0.154	0 (0.0%)
Serious morbidity (composite)	1317/4587 (28.7%)	48/172 (27.9%)	8/35 (22.9%)	0.730	0 (0.0%)
30‐day mortality	44/4587 (1.0%)	1/172 (0.6%)	0/35 (0.0%)	0.807	0 (0.0%)
Readmission	858/4587 (18.7%)	28/172 (16.3%)	5/35 (14.3%)	0.584	0 (0.0%)
Unplanned readmission	857/4587 (18.7%)	28/172 (16.3%)	5/35 (14.3%)	0.588	0 (0.0%)
Reoperation	174/4587 (3.8%)	6/172 (3.5%)	2/35 (5.7%)	0.820	0 (0.0%)
**Individual complications**					
Superficial incisional SSI	132/4587 (2.9%)	4/172 (2.3%)	0/35 (0.0%)	0.614	0 (0.0%)
Deep incisional SSI	7/4587 (0.2%)	0/172 (0.0%)	0/35 (0.0%)	1.000	0 (0.0%)
Organ/space SSI	754/4587 (16.4%)	25/172 (14.5%)	3/35 (8.6%)	0.370	0 (0.0%)
Wound dehiscence	24/4587 (0.5%)	0/172 (0.0%)	0/35 (0.0%)	0.691	0 (0.0%)
Pneumonia	134/4587 (2.9%)	7/172 (4.1%)	2/35 (5.7%)	0.435	0 (0.0%)
Unplanned reintubation	77/4587 (1.7%)	3/172 (1.7%)	1/35 (2.9%)	1.000	0 (0.0%)
Pulmonary embolism	90/4587 (2.0%)	5/172 (2.9%)	1/35 (2.9%)	0.584	0 (0.0%)
Ventilator > 48 h	53/4587 (1.2%)	2/172 (1.2%)	1/35 (2.9%)	0.813	0 (0.0%)
Acute renal failure/dialysis	31/4587 (0.7%)	0/172 (0.0%)	0/35 (0.0%)	0.538	0 (0.0%)
Urinary tract infection	72/4587 (1.6%)	5/172 (2.9%)	1/35 (2.9%)	0.296	0 (0.0%)
Stroke/CVA	4/4587 (0.1%)	0/172 (0.0%)	0/35 (0.0%)	1.000	0 (0.0%)
Cardiac arrest requiring CPR	34/4587 (0.7%)	2/172 (1.2%)	0/35 (0.0%)	0.726	0 (0.0%)
Myocardial infarction	30/4587 (0.7%)	1/172 (0.6%)	0/35 (0.0%)	1.000	0 (0.0%)
Bleeding requiring transfusion	393/4587 (8.6%)	34/172 (19.8%)	8/35 (22.9%)	< 0.001	0 (0.0%)
Venous thrombosis requiring therapy	121/4587 (2.6%)	5/172 (2.9%)	2/35 (5.7%)	0.604	0 (0.0%)
Sepsis	229/4587 (5.0%)	7/172 (4.1%)	1/35 (2.9%)	0.731	0 (0.0%)
Septic shock	75/4587 (1.6%)	3/172 (1.7%)	1/35 (2.9%)	1.000	0 (0.0%)
Clostridioides difficile colitis	26/4587 (0.6%)	0/172 (0.0%)	0/35 (0.0%)	0.696	0 (0.0%)
**Pancreas‐specific**					
Postoperative pancreatic fistula, ISGPS grade B/C	844/4550 (18.5%)	27/170 (15.9%)	5/35 (14.3%)	0.555	39 (0.8%)
Delayed gastric emptying	410/4576 (9.0%)	18/171 (10.5%)	4/35 (11.4%)	0.691	12 (0.3%)
Post‐pancreatectomy hemorrhage (PUF 2021–2023 only)	179/2879 (6.2%)	7/95 (7.4%)	6/23 (26.1%)	< 0.001	3 (0.1%)
Postoperative acute pancreatitis, grade B/C (PUF 2019–2020 only)	209/1687 (12.4%)	2/75 (2.7%)	0/12 (0.0%)	0.017	20 (1.1%)

*Note:* Data are n/N (%), where N is the number of patients with the variable recorded, or median (interquartile range). Continuous variables were compared with the Kruskal–Wallis test; categorical variables with the chi‐square test, Fisher exact test, or a Monte Carlo Fisher–Freeman–Halton test where cell counts were sparse. Composite morbidity was constructed only from NSQIP fields collected in every study year. Post‐pancreatectomy hemorrhage and postoperative acute pancreatitis are reported only for the PUF years in which those fields were collected, with the corresponding denominators shown. Pancreatic fistula and postoperative acute pancreatitis are ISGPS grade B/C.

Abbreviations: SSI, surgical site infection; ISGPS, International Study Group of Pancreatic Surgery

### 3.3 Multivariable Analyses

After adjustment, neither concurrent hepatectomy (OR 1.02, 95% CI 0.71–1.48) nor concurrent ablation (OR 0.94, 95% CI 0.46–1.93) was associated with 30‐day morbidity (Table 4; Supporting Information S1: Figure 1A). Pancreatoduodenectomy was associated with morbidity (OR 2.38, 95% CI 2.05–2.76) and a minimally invasive approach with lower odds of morbidity (OR 0.69, 95% CI 0.60–0.80). Findings were similar for serious morbidity (concurrent hepatectomy OR 0.84, 95% CI 0.56–1.26).

**Table 4:** Multivariable logistic regression analysis of predictors of 30‐day overall morbidity.

Variable	OR (95% CI)	*p* value
**Study group**		
Reference: isolated pancreatectomy		
+ Hepatectomy	1.02 (0.71 – 1.48)	0.896
+ Ablation only	0.94 (0.46 – 1.93)	0.862
Age (per year)	1.00 (0.99 – 1.00)	0.679
Male sex	1.26 (1.12 – 1.43)	< 0.001
Body mass index (per kg/m^2^)	1.03 (1.02 – 1.04)	< 0.001
ASA class III–V	1.17 (1.01 – 1.35)	0.040
Dependent functional status	1.58 (0.74 – 3.36)	0.237
Preoperative albumin (per g/dL)	0.76 (0.65 – 0.88)	< 0.001
Preoperative bilirubin (per mg/dL)	0.99 (0.91 – 1.07)	0.780
Chemotherapy within 90 days	1.25 (0.92 – 1.69)	0.155
Pancreatoduodenectomy (vs. distal)	2.38 (2.05 – 2.76)	< 0.001
Other pancreatectomy (vs. distal)	0.95 (0.79 – 1.14)	0.575
Minimally invasive (vs. open)	0.69 (0.60 – 0.80)	< 0.001
Approach not recorded (vs. open)	0.82 (0.62 – 1.07)	0.148
Pathologic M1 (vs. M0/Mx)	1.14 (0.85 – 1.54)	0.378
M stage not recorded (vs. M0/Mx)	0.95 (0.82 – 1.10)	0.478
Functioning (vs. non‐functioning)	0.92 (0.79 – 1.07)	0.286
Benign/no malignant histology (vs. non‐functioning)	0.72 (0.58 – 0.91)	0.005
PUF year 2020 (vs. 2019)	1.03 (0.85 – 1.26)	0.755
PUF year 2021 (vs. 2019)	1.17 (0.92 – 1.47)	0.198
PUF year 2022 (vs. 2019)	0.95 (0.78 – 1.15)	0.583
PUF year 2023 (vs. 2019)	1.07 (0.88 – 1.29)	0.522

*Note*: Odds ratios and 95% confidence intervals from multivariable logistic regression with prespecified covariates. The study‐group term was tested as a block by likelihood‐ratio test (2 df, *p* = 0.973). Model *n* = 4794, events = 1874.

Concurrent liver‐directed surgery was not associated with readmission (Table 5), reoperation (Table 6), or mortality (Table 7); adjusted odds ratios for both concurrent groups across all five outcomes are displayed in Supporting Information S1: Figure 1A. Pancreatoduodenectomy was independently associated with readmission (OR 1.76, 95% CI 1.47–2.11), reoperation (OR 2.16, 95% CI 1.52–3.08), and mortality (OR 2.50, 95% CI 1.39–4.50); adjusted estimates for type of pancreatic resection and operative approach are summarized in Supporting Information S1: Figure 1B. The null association between concurrent liver‐directed surgery and morbidity persisted in every prespecified sensitivity analysis, including inclusion of the unlisted liver codes within the hepatectomy group, restriction to M1 disease, complete‐case analysis, and multiple imputation (Supporting Information S2: Table 2).

**Table 5:** Multivariable logistic regression analysis of predictors of 30‐day readmission.

Variable	OR (95% CI)	*p* value
**Study group**		
Reference: isolated pancreatectomy		
+ Hepatectomy	0.81 (0.50 – 1.30)	0.379
+ Ablation only	0.74 (0.28 – 2.00)	0.557
Age (per year)	0.99 (0.99 – 1.00)	0.005
Male sex	1.25 (1.07 – 1.45)	0.004
Body mass index (per kg/m^2^)	1.03 (1.02 – 1.04)	< 0.001
ASA class III–V	1.05 (0.88 – 1.26)	0.587
Dependent functional status	1.59 (0.67 – 3.79)	0.296
Preoperative albumin (per g/dL)	0.99 (0.82 – 1.19)	0.926
Preoperative bilirubin (per mg/dL)	0.87 (0.76 – 0.99)	0.037
Chemotherapy within 90 days	1.15 (0.80 – 1.66)	0.448
Pancreatoduodenectomy (vs. distal)	1.76 (1.47 – 2.11)	< 0.001
Other pancreatectomy (vs. distal)	1.07 (0.86 – 1.34)	0.542
Minimally invasive (vs. open)	0.90 (0.75 – 1.07)	0.239
Approach not recorded (vs. open)	0.73 (0.52 – 1.02)	0.061
Pathologic M1 (vs. M0/Mx)	0.90 (0.62 – 1.31)	0.583
M stage not recorded (vs. M0/Mx)	0.90 (0.76 – 1.08)	0.263
Functioning (vs. non‐functioning)	0.95 (0.78 – 1.15)	0.576
Benign/no malignant histology (vs. non‐functioning)	0.90 (0.68 – 1.18)	0.449
PUF year 2020 (vs. 2019)	0.98 (0.76 – 1.25)	0.841
PUF year 2021 (vs. 2019)	1.25 (0.94 – 1.65)	0.120
PUF year 2022 (vs. 2019)	1.02 (0.81 – 1.29)	0.869
PUF year 2023 (vs. 2019)	1.07 (0.85 – 1.36)	0.556

*Note:* Odds ratios and 95% confidence intervals from multivariable logistic regression with prespecified covariates. The study‐group term was tested as a block by likelihood‐ratio test (2 df, *p* = 0.598). Model *n* = 4794, events = 891.

**Table 6:** Multivariable logistic regression analysis of predictors of 30‐day reoperation.

Variable	OR (95% CI)	*p* value
**Study group**		
Reference: isolated pancreatectomy		
+ Hepatectomy	0.87 (0.35 – 1.80)	0.730
+ Ablation only	1.81 (0.36 – 5.55)	0.411
Age (per year)	1.01 (0.99 – 1.02)	0.377
ASA class III–V	1.14 (0.79 – 1.66)	0.494
Pancreatoduodenectomy (vs. distal)	2.16 (1.52 – 3.08)	< 0.001
Other pancreatectomy (vs. distal)	1.20 (0.73 – 1.90)	0.458
Minimally invasive (vs. open)	0.70 (0.48 – 1.02)	0.063
Approach not recorded (vs. open)	0.99 (0.60 – 1.56)	0.955

*Note:* Odds ratios and 95% confidence intervals from multivariable logistic regression with prespecified covariates. The study‐group term was tested as a block by likelihood‐ratio test (2 df, *p* = 0.224). Model n = 4794, events = 182. Estimated by Firth penalized likelihood with profile‐likelihood confidence intervals because of the small number of events in the ablation group.

**Table 7:** Multivariable logistic regression analysis of predictors of 30‐day mortality.

Variable	OR (95% CI)	*p* value
**Study group**		
Reference: isolated pancreatectomy		
+ Hepatectomy	0.89 (0.10–3.34)	0.882
+ Ablation only	1.58 (0.01–11.70)	0.768
Age (per year)	1.02 (1.00–1.05)	0.047
ASA class III–V	2.09 (0.92–5.80)	0.083
Pancreatoduodenectomy (vs. other resection)	2.50 (1.39–4.50)	0.002

*Note:* Odds ratios and 95% confidence intervals from multivariable logistic regression with prespecified covariates. The study‐group term was tested as a block by likelihood‐ratio test (2 df, *p* = 1.000). Model *n* = 4794, events = 45. Estimated by Firth penalized likelihood with profile‐likelihood confidence intervals. The model was limited to five parameters given 45 events.

## 4 Discussion

This study demonstrates that pancreatectomy with concurrent liver‐directed surgery for pancreatic neuroendocrine tumors is not associated with worse short‐term outcomes than pancreatectomy alone. To our knowledge, this is the largest study to specifically examine this comparison, providing a contemporary multicenter assessment of perioperative risk [11, 12, 13].

An important and, to our knowledge, previously unreported observation is that concurrent liver‐directed surgery for pNETs in contemporary national practice is parenchymal‐sparing. Across 5 years of NSQIP data, fewer than 10 patients underwent a formal major hepatectomy concurrent with pancreatectomy. This is consistent with the evolution toward parenchymal‐preserving strategies for neuroendocrine liver metastases, in which wedge resection, enucleation, and ablation are favored in order to preserve functional liver volume and permit repeat intervention for the recurrent disease that is expected in this population.

Prior studies, such as Kone et al., have analyzed patients undergoing isolated pancreatectomy, isolated hepatectomy, and concurrent pancreatectomy with hepatectomy [10]. Our analysis extends these findings using a larger, nationally representative cohort with standardized perioperative outcome reporting and explicit procedural definitions. On multivariable analysis, the addition of a concurrent liver‐directed procedure was not independently associated with 30‐day morbidity, readmission, reoperation, or mortality, indicating that perioperative risk in this population is driven principally by the pancreatic operation rather than the hepatic component. The one consistent exception was transfusion, which was more than twice as common when a liver‐directed procedure was performed. This is a clinically coherent finding and is a reasonable focus of preoperative planning, including blood availability and intraoperative blood‐conservation strategies, in patients being considered for a combined operation.

While these data support the perioperative safety of concurrent resection, they do not address whether concurrent surgery is oncologically equivalent or superior to a staged approach. The NCCN Neuroendocrine and Adrenal Tumors guideline recommends ‘staged or synchronous resection when possible,’ noting that when performing staged pancreatoduodenectomy and liver resection, hepatectomy prior to pancreatic resection should be considered to reduce the risk of perihepatic sepsis [13]. Gudmundsdottir et al. reported similar 5‐year overall survival between combined pancreatic‐hepatic resection (64%) and isolated hepatectomy (65%, *p* = 0.93) at a single high‐volume institution [11]. Pu et al. demonstrated that primary resection with metastasectomy was associated with a survival advantage compared with no surgical intervention for pNETs with synchronous liver metastases (HR 0.22–0.47, *p* < 0.001) [14]. However, no randomized data directly compare concurrent versus staged approaches, and the optimal timing of hepatic resection relative to pancreatectomy remains an important unanswered question.

Pancreatoduodenectomy was the dominant determinant of adverse outcome in every model, being associated with morbidity, readmission, reoperation, and mortality. This is consistent with a substantial existing literature and reinforces that, in patients with pNET and hepatic metastases, the morbidity of the operation is largely attributable to the pancreatic resection required rather than to the addition of liver‐directed surgery [15].

In our study, minimally invasive approaches were more commonly used for isolated pancreatectomy. A minimally invasive approach was associated with lower odds of morbidity. This association should be interpreted with caution: in a retrospective registry study, patients selected for a minimally invasive approach differ systematically from those selected for an open operation in tumor size, location, extent of disease, and the need for concurrent procedures, and residual confounding by indication cannot be excluded. The LEOPARD trial, the largest randomized controlled trial comparing minimally invasive versus open distal pancreatectomy for isolated left‐sided pancreatic tumors, found no differences in morbidity or mortality, but showed faster functional recovery with minimally invasive surgery [16]. Notably, the LEOPARD trial enrolled only patients undergoing isolated distal pancreatectomy and excluded those with concurrent hepatic procedures, limiting its direct applicability to the combined resection population studied here.

Patients with non‐functioning pancreatic NETs are often discovered later at higher grades and larger sizes and may have worse oncologic prognosticators, such as lymphovascular or perineural invasion, than functional pNETs [17, 18]. Age is an important prognostic factor for overall survival in pNETs [5, 19, 20]. As such, age‐related mortality may reflect the natural history of these tumors and competing risk as much as the outcome of surgical intervention.

### 4.1 Limitations

This is a retrospective observational analysis and the associations reported should not be interpreted as causal [21]. NSQIP captures 30‐day outcomes only and contains no data on the number, size, or distribution of liver lesions, the completeness of cytoreduction, tumor grade or Ki‐67, or long‐term oncologic outcome, so residual confounding by hepatic disease burden cannot be excluded; pathologic M stage, which we used as a proxy, was not recorded in 32.2% of the cohort. Operative approach was not recorded in 10.3%. Liver‐directed procedures captured only by unlisted CPT codes were excluded from the primary analysis, and intraoperative ablation performed without a separate CPT claim would not be identifiable in these data, so the true frequency of concurrent ablation is likely underestimated. NSQIP does not capture whether preoperative or perioperative somatostatin analog therapy was administered, which may influence both tumor biology and perioperative outcomes, particularly pancreatic fistula rates [22]. Finally, formal major hepatectomy was performed in only nine patients, and this study is not powered to assess its safety.

## 5 Conclusion

Concurrent liver‐directed surgery at the time of pancreatectomy for pancreatic neuroendocrine tumors was not associated with increased 30‐day morbidity, readmission, reoperation, or mortality. In contemporary national practice, this surgery is almost entirely parenchymal‐sparing, and perioperative risk appears to be determined principally by the pancreatic resection performed.

## Funding

The authors have nothing to report.

## Ethics Statement

All procedures performed in this study involving human participants were in accordance with the ethical standards of the institutional and/or national research committee. All procedures were in accordance with the 1964 Helsinki Declaration and its later amendments or comparable ethical standards.

## Conflicts of Interest

The authors declare no conflicts of interest.

## 6 Synopsis

Using ACS‐NSQIP, we evaluated whether concurrent liver‐directed surgery at the time of pancreatectomy for pNETs increases short‐term perioperative risk. Concurrent liver‐directed surgery was not associated with a higher rate of morbidity, reoperation, readmission, or mortality; perioperative risk was driven by the pancreatic operation itself.

## Supporting information


Supporting File 1

Supporting File 2

## Data Availability

The data that support the findings of this study are available from the corresponding author upon reasonable request.
