# Supplementary material for: Perioperative Risks of Concurrent Pancreatic and Hepatic Resections for Neuroendocrine Tumors
Source: J Surg Oncol. 2026 Aug 6;134(6):830–9. doi: 10.1002/jso.70352 (PMC13732549; doi:10.1002/jso.70352)
Supplement: Supplementary file 1 [file JSO-134-830-s001.docx]

**Supplementary Table S1. CPT Codes Defining Each Liver-Directed Procedure Group**

| **Analytic group** | **CPT code** | **CPT descriptor** | **n in full extract (any position)** |
| --- | --- | --- | --- |
| Major hepatectomy | 47122 | Hepatectomy, trisegmentectomy | 3 |
| Major hepatectomy | 47125 | Hepatectomy, total left lobectomy | 3 |
| Major hepatectomy | 47130 | Hepatectomy, total right lobectomy | 3 |
| Minor / parenchymal-sparing hepatectomy | 47120 | Hepatectomy, partial lobectomy (wedge/segmental) | 163 |
| Ablation | 47370 | Laparoscopic ablation of liver tumor(s), radiofrequency | 8 |
| Ablation | 47371 | Laparoscopic ablation of liver tumor(s), cryosurgical | 0 |
| Ablation | 47380 | Open ablation of liver tumor(s), radiofrequency | 60 |
| Ablation | 47381 | Open ablation of liver tumor(s), cryosurgical | 2 |
| Ablation | 47382 | Percutaneous ablation of liver tumor(s), radiofrequency | 5 |
| Ablation | 47383 | Percutaneous ablation of liver tumor(s), microwave | 0 |
| Unlisted liver procedure (excluded from primary) | 47379 | Unlisted laparoscopic procedure, liver | 45 |
| Unlisted liver procedure (excluded from primary) | 47399 | Unlisted procedure, liver | 23 |
| Diagnostic liver biopsy (not liver-directed therapy) | 47100 | Biopsy of liver, wedge | 161 |

*CPT 49000 (exploratory laparotomy) and CPT 47100 (wedge liver biopsy) were not treated as liver-directed therapeutic procedures. Counts are the number of patients in the full extract carrying that code in any procedure field.*

**Supplementary Table S2. Sensitivity Analyses for the Study-Group Effect**

| **Analysis** | **Study arm** | **Adjusted OR (95% CI)** | **P-value** | **n** | **Events** | **Definition** |
| --- | --- | --- | --- | --- | --- | --- |
| Primary analysis — any morbidity | Concurrent hepatectomy | 1.02 (0.71 – 1.48) | 0.896 | 4794 | 1874 | Missing-indicator model, full cohort |
| Primary analysis — any morbidity | Concurrent ablation only | 0.94 (0.46 – 1.93) | 0.862 | 4794 | 1874 | Missing-indicator model, full cohort |
| Primary analysis — serious morbidity | Concurrent hepatectomy | 0.84 (0.56 – 1.26) | 0.406 | 4794 | 1373 | Missing-indicator model, full cohort |
| Primary analysis — serious morbidity | Concurrent ablation only | 0.69 (0.30 – 1.60) | 0.387 | 4794 | 1373 | Missing-indicator model, full cohort |
| Sensitivity (a) — any morbidity | Concurrent hepatectomy | 1.07 (0.78 – 1.46) | 0.694 | 4859 | 1901 | Unlisted liver CPT codes (47379/47399) included in the hepatectomy arm |
| Sensitivity (a) — any morbidity | Concurrent ablation only | 0.94 (0.46 – 1.94) | 0.876 | 4859 | 1901 | Unlisted liver CPT codes (47379/47399) included in the hepatectomy arm |
| Sensitivity (a) — serious morbidity | Concurrent hepatectomy | 0.83 (0.58 – 1.17) | 0.282 | 4859 | 1389 | Unlisted liver CPT codes (47379/47399) included in the hepatectomy arm |
| Sensitivity (a) — serious morbidity | Concurrent ablation only | 0.68 (0.30 – 1.57) | 0.365 | 4859 | 1389 | Unlisted liver CPT codes (47379/47399) included in the hepatectomy arm |
| Sensitivity (b) — any morbidity | Concurrent hepatectomy | 1.07 (0.62 – 1.85) | 0.812 | 303 | 139 | Restricted to pathologic M1 disease |
| Sensitivity (b) — any morbidity | Concurrent ablation only | 0.53 (0.19 – 1.45) | 0.216 | 303 | 139 | Restricted to pathologic M1 disease |
| Sensitivity (b) — serious morbidity | Concurrent hepatectomy | 0.86 (0.48 – 1.53) | 0.609 | 303 | 88 | Restricted to pathologic M1 disease |
| Sensitivity (b) — serious morbidity | Concurrent ablation only | 0.34 (0.09 – 1.29) | 0.113 | 303 | 88 | Restricted to pathologic M1 disease |
| Sensitivity (d1) — any morbidity | Concurrent hepatectomy | 0.97 (0.63 – 1.48) | 0.879 | 3436 | 1354 | Complete-case analysis |
| Sensitivity (d1) — any morbidity | Concurrent ablation only | 0.59 (0.26 – 1.34) | 0.209 | 3436 | 1354 | Complete-case analysis |
| Sensitivity (d1) — serious morbidity | Concurrent hepatectomy | 0.81 (0.51 – 1.30) | 0.385 | 3436 | 981 | Complete-case analysis |
| Sensitivity (d1) — serious morbidity | Concurrent ablation only | 0.58 (0.22 – 1.49) | 0.255 | 3436 | 981 | Complete-case analysis |
| Sensitivity (d2) — any morbidity | Concurrent hepatectomy | 1.02 (0.70 – 1.48) | 0.919 | 4794 | 1874 | Multiple imputation (m=10), pooled by Rubin's rules |
| Sensitivity (d2) — any morbidity | Concurrent ablation only | 0.96 (0.47 – 1.97) | 0.912 | 4794 | 1874 | Multiple imputation (m=10), pooled by Rubin's rules |
| Sensitivity (d2) — serious morbidity | Concurrent hepatectomy | 0.85 (0.56 – 1.27) | 0.419 | 4794 | 1373 | Multiple imputation (m=10), pooled by Rubin's rules |
| Sensitivity (d2) — serious morbidity | Concurrent ablation only | 0.71 (0.31 – 1.63) | 0.418 | 4794 | 1373 | Multiple imputation (m=10), pooled by Rubin's rules |

*All models are adjusted as in Table 4. The association between concurrent liver-directed surgery and morbidity was null in every specification.*

**Supplementary Table S3. Outcome Definitions and PUF-Year Availability**

| **Outcome** | **NSQIP field** | **PUF years available** | **In 'any morbidity' composite** | **In 'serious morbidity' composite** |
| --- | --- | --- | --- | --- |
| Superficial incisional SSI | SUPINFEC | 2019–2023 | Yes | No |
| Deep incisional SSI | WNDINFD | 2019–2023 | Yes | Yes |
| Organ/space SSI | ORGSPCSSI | 2019–2023 | Yes | Yes |
| Wound dehiscence | DEHIS | 2019–2023 | Yes | Yes |
| Pneumonia | OUPNEUMO | 2019–2023 | Yes | Yes |
| Unplanned reintubation | REINTUB | 2019–2023 | Yes | Yes |
| Pulmonary embolism | PULEMBOL | 2019–2023 | Yes | Yes |
| Ventilator > 48 h | FAILWEAN | 2019–2023 | Yes | Yes |
| Acute renal failure / dialysis | OPRENAFL | 2019–2023 | Yes | Yes |
| Urinary tract infection | URNINFEC | 2019–2023 | Yes | No |
| Stroke / CVA | CNSCVA | 2019–2023 | Yes | Yes |
| Cardiac arrest requiring CPR | CDARREST | 2019–2023 | Yes | Yes |
| Myocardial infarction | CDMI | 2019–2023 | Yes | Yes |
| Bleeding requiring transfusion | OTHBLEED | 2019–2023 | Yes | No |
| Venous thrombosis requiring therapy | OTHDVT | 2019–2023 | Yes | No |
| Sepsis | OTHSYSEP | 2019–2023 | Yes | Yes |
| Septic shock | OTHSESHOCK | 2019–2023 | Yes | Yes |
| Clostridioides difficile colitis | OTHCDIFF | 2019–2023 | Yes | Yes |
| Pancreatic fistula, ISGPS grade B/C | PAN_FISTULA | 2019–2023 | Yes | Yes |
| Delayed gastric emptying | PAN_DELGASTRIC_20140315 | 2019–2023 | Yes | No |
| Post-pancreatectomy hemorrhage | PAN_HEMORRHAGE | 2021–2023 | No (year-restricted) | No |
| Postoperative acute pancreatitis, grade B/C | PAN_POAP | 2019–2020 | No (year-restricted) | No |
| Renal insufficiency | RENAINSF | 2019, 2020, 2022, 2023 | No (not collected in 2021) | No |

*Renal insufficiency (RENAINSF) is not collected in the 2021 PUF and was therefore excluded from the composite; acute renal failure and postoperative dialysis (OPRENAFL), collected in all years, were retained.*

**Supplementary Table S4. Missing and Not-Recorded Data in the Analytic Cohort**

| **Variable** | **Missing / not recorded, n** | **% of analytic cohort** |
| --- | --- | --- |
| Age | 0 | 0.0% |
| Body mass index | 30 | 0.6% |
| ASA class | 4 | 0.1% |
| Functional status | 4 | 0.1% |
| Preoperative albumin | 835 | 17.4% |
| Preoperative bilirubin | 724 | 15.1% |
| Chemotherapy within 90 days | 30 | 0.6% |
| Operative approach | 496 | 10.3% |
| Pathologic M stage | 1545 | 32.2% |
| Any morbidity (composite) | 0 | 0.0% |
| Serious morbidity (composite) | 0 | 0.0% |
| Pancreatic fistula | 39 | 0.8% |
| Delayed gastric emptying | 12 | 0.3% |
| 30-day mortality | 0 | 0.0% |
| Readmission | 0 | 0.0% |
| Reoperation | 0 | 0.0% |

*Missingness was handled in the primary models by explicit indicator or category so that the full cohort was retained; complete-case and multiply imputed analyses are shown in Supplementary Table S2.*
