# Supplementary material for: Perioperative Risks of Concurrent Pancreatic and Hepatic Resections for Neuroendocrine Tumors
Source: J Surg Oncol. 2026 Aug 6;134(6):830–9. doi: 10.1002/jso.70352 (PMC13732549; doi:10.1002/jso.70352)
Supplement: Supplementary file 2 [file JSO-134-830-s002.docx]

**Supplementary Figures**

**Supplementary Figure S1. Adjusted odds ratios from the multivariable models. (A) Concurrent liver-directed surgery across all 30-day outcomes. (B) Type of pancreatic resection and operative approach.**

Squares are adjusted odds ratios and horizontal lines are 95% confidence intervals; the x-axis is logarithmic and the vertical dashed line marks the null value of 1.0. Panel A shows concurrent hepatectomy (n = 172) and concurrent ablation alone (n = 35), each compared with isolated pancreatectomy (n = 4,587), for all five 30-day outcomes (Tables 4–7 and Supplementary Table S2); arrowheads denote confidence limits extending beyond the plotted range. The confidence interval crosses 1.0 for every estimate, and the study-group term was not significant by likelihood-ratio test for any outcome. Panel B shows the operative factors from the models for any morbidity (Table 4), readmission (Table 5), and reoperation (Table 6). Thirty-day mortality is not shown in Panel B because the reduced mortality model (Table 7) used a different reference category for type of resection and did not include operative approach. All models are adjusted for the prespecified covariate set described in the Methods; reoperation and mortality were estimated by Firth penalized likelihood. The associations shown for a minimally invasive approach should be interpreted as associations only, given the potential for confounding by indication.

**Supplementary Figure S2. Observed 30-day outcomes by study group.**

Bars are observed event rates and error bars are 95% Wilson score confidence intervals, calculated from the counts and denominators reported in Table 3. Confidence intervals overlap across all three groups for every outcome, and no comparison reached statistical significance (any morbidity P = 0.154; serious morbidity P = 0.730; readmission P = 0.584; reoperation P = 0.820; 30-day mortality P = 0.807). The width of the intervals for the concurrent ablation group reflects its small size (n = 35) and illustrates the limited precision available for that comparison; no deaths occurred in that group.

**Supplementary Figure S3. Observed rates of individual and pancreas-specific complications by study group.**

Bars are observed event rates and error bars are 95% Wilson score confidence intervals, calculated from the counts and denominators reported in Table 3. Bleeding requiring transfusion was the only complication that differed significantly between groups (8.6%, 19.8%, and 22.9%; P < 0.001). Post-pancreatectomy hemorrhage (PPH) is collected only in the 2021–2023 files and postoperative acute pancreatitis (POAP) only in the 2019–2020 files, and each is therefore plotted against its year-restricted denominator; the corresponding group denominators are 2,879 / 95 / 23 for PPH and 1,687 / 75 / 12 for POAP. The apparent excess of PPH in the ablation group rests on 6 events among 23 patients and should be interpreted with caution. Pancreatic fistula and postoperative acute pancreatitis are ISGPS grade B/C. SSI = surgical site infection; POPF = postoperative pancreatic fistula; ISGPS = International Study Group of Pancreatic Surgery.
